# 98729 Professional Development Core of the Hispanic Alliance for Clinical and Translational Research: a scientific productivity catalyst for underrepresented minorities (URM) in Clinical and Translational Research (CTR)

**DOI:** 10.1017/cts.2021.570

**Published:** 2021-03-30

**Authors:** Mariela Torres-Cintrón, Margarita Irizarry-Ramírez, Harold Saavedra

**Affiliations:** 1 Hispanic Alliance for Clinical and Translational Research, University of Puerto Rico Medical Sciences Campus; 2 Hispanic Alliance for Clinical and Translational Research, Ponce Health Sciences University

## Abstract

ABSTRACT IMPACT: The Hispanic Alliance for Clinical and Translational Research Professional Development Core (PDC) will contribute to the improvement of the health of an increasing US Hispanic population, by supporting and training a new cadre of Hispanic/Latino CTR researchers and community leaders that understand this population’s prevalent health needs. OBJECTIVES/GOALS: To use the Professional Development Core (PDC) of the Hispanic Alliance for Clinical and Translational Research (Alliance) as a hub that coordinates training, mentoring programs, and grant support to address the need for more underrepresented minorities (URM) in clinical and translational research and mentoring. METHODS/STUDY POPULATION: PDC will: (1). Coordinate and offer an effective educational program based for new and mid-career researchers to address the gaps in research competencies on Hispanic/Latino health and healthcare through web-based asynchronous distance training, enhanced with face-to-face interactions. (2). Establish a robust mentoring program to address the mentoring gap for URM faculty by developing mentorship skills of faculty and researchers through a variety of resources, and offering protected time to mentor-mentee teams. (3). Design and implement a tailor-made curriculum to train scientists and community partners jointly, enabling them to carry out multidisciplinary research responsive to the Hispanic/Latino community health’s needs. RESULTS/ANTICIPATED RESULTS: From 2010 to 2019 the PDC supported over 1,000 researchers and faculty and provided 52 activities over the 9 years. PDC-supported researchers submitted 56 proposals and 21 grants (37.5.%) were awarded, for a total of $2, 225,751.00, and to published 94 peer-review papers. We expect that through Alliance PDC will sponsor at least 20 new trainees/mentees in Clinical and Translational Research (CTR), 20 new certified mentors, a continuous support program, and an increase of 30% in the scientific productivity (e.g., grants submission and peer-reviewed publications) of the Hispanic CTRs in Puerto Rico and the establishment of long-term links with the Hispanic community in Puerto Rico and across the United States to address its health needs. DISCUSSION/SIGNIFICANCE OF FINDINGS: The PDC programs are significant in addressing the need for qualified researchers and mentors that understand, have the know-how, and are interested in addressing the health needs of a growing USA Hispanic medically underserved population.

